# Supplementary material for: Integration of Radiometric Ground-Based Data and High-Resolution QuickBird Imagery with Multivariate Modeling to Estimate Maize Traits in the Nile Delta of Egypt
Source: Sensors (Basel). 2021 Jun 6;21(11):3915. doi: 10.3390/s21113915 (PMC8200994; doi:10.3390/s21113915)
Supplement: Supplementary file 1 [file sensors-21-03915-s001.zip › sensors-1209459-supplementary.pdf]

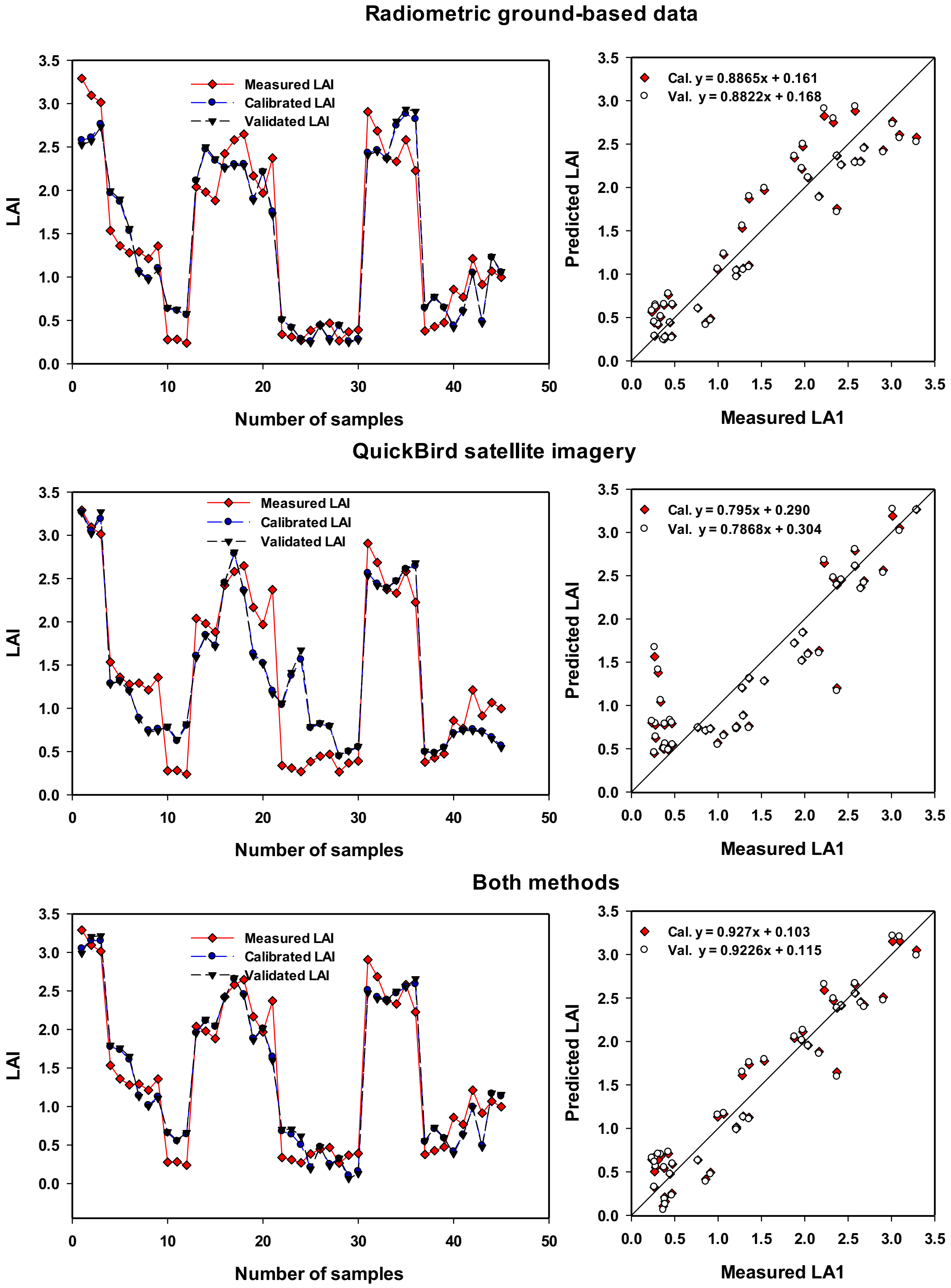

Figure S1. Comparison between measuring series, calibrating series and validating series for leaf area index (LAI) of maize using the MLR models based on selected vegetation-SRIs extracted from (a) radiometric ground-based data, (b) extracted from QuickBird satellite imagery and (c) extracted from both methods.

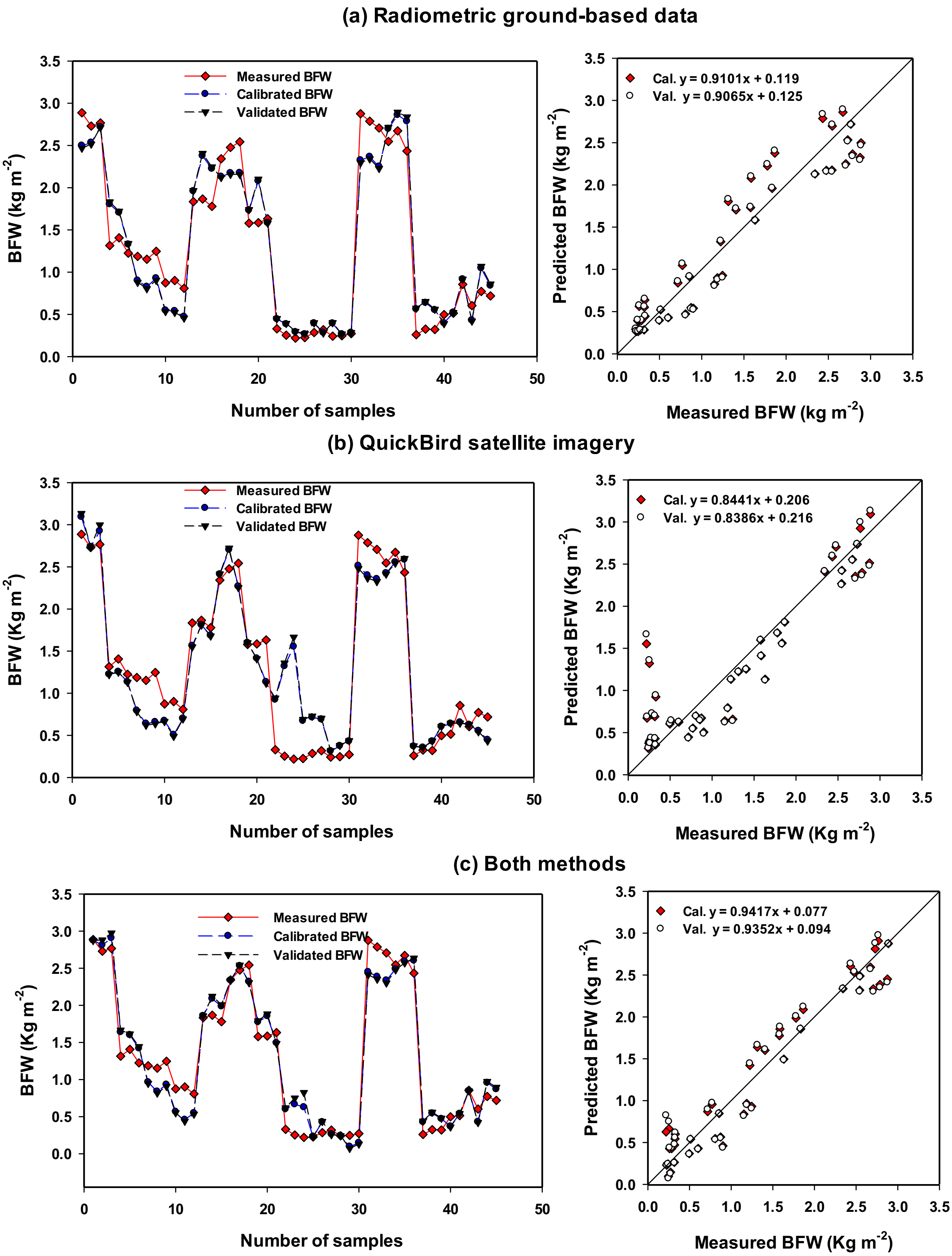

Figure S2. Comparison between measuring series, calibrating series and validating series for biomass fresh weight (BFW) of maize using the MLR models based on selected vegetation-SRIs extracted from (a) radiometric ground-based data, (b) extracted from QuickBird satellite imagery and (c) extracted from both methods.

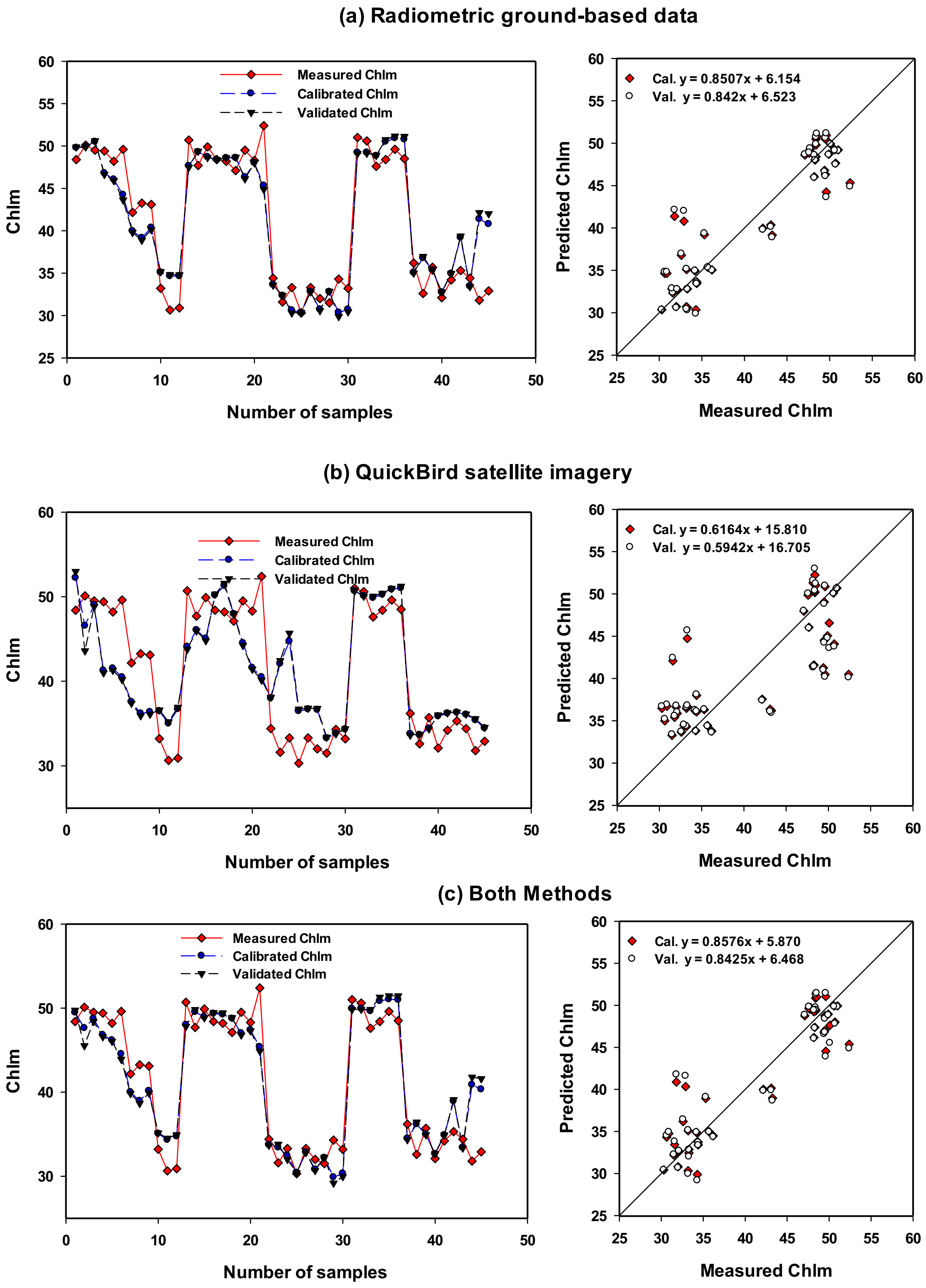

Figure S3. Comparison between measuring series, calibrating series and validating series for Chlorophll meter (Chlm) of maize using the MLR models based on selected vegetation-SRIs extracted from (a) radiometric ground-based data, (b) extract from QuickBird satellite imagery and (c) extracted from both methods.
